# Development and use of a polarized equine upper respiratory tract mucosal explant system to study the early phase of pathogenesis of a European strain of equine arteritis virus

**DOI:** 10.1186/1297-9716-44-22

**Published:** 2013-03-28

**Authors:** Sabrina Vairo, Wim Van den Broeck, Herman Favoreel, Alessandra Scagliarini, Hans Nauwynck

**Affiliations:** 1 Laboratory of Virology, Department of Virology, Parasitology and Immunology, Faculty of Veterinary Medicine, Ghent University, Merelbeke B-9820, Belgium; 2 Department of Morphology, Faculty of Veterinary Medicine, Ghent University, Merelbeke, B-9820, Belgium; 3 Laboratory of Immunology, Department of Virology, Parasitology and Immunology, Faculty of Veterinary Medicine, Ghent University, Merelbeke, B-9820, Belgium; 4 Department of Veterinary Medical Science, Alma Mater Studiorum, Bologna University, Ozzano dell’Emilia, Italy

## Abstract

The upper respiratory tract mucosa represents the first line of defense, which has to be overcome by pathogens before invading the host. Considering the economic and ethical aspects involved in using experimental animals for pathogenesis studies, respiratory mucosal explants, in which the tissue’s three-dimensional architecture is preserved, may be ideal alternatives. Different respiratory mucosal explant cultures have been developed. However, none of them could be inoculated with pathogens solely at the epithelium side. In the present study, equine nasal and nasopharyngeal explants were embedded in agarose (3%), leaving the epithelium side exposed to allow apical inoculation. Morphometric analysis did not show degenerative changes during 72 h of cultivation. The number of apoptotic cells in the mucosa slightly increased over time. After validation, the system was used for apical infection with a European strain (*08P178*) of equine arteritis virus (EAV) (10^7.6^TCID_50_/mL per explant). Impermeability of agarose to virus particles was demonstrated by the absence of labeled microspheres (40nm) and a lack of EAV-antigens in RK13 cells seeded underneath the agarose layer in which inoculated explants were embedded. At 72 hpi, 27% of the EAV-positive cells were CD172a^+^ and 19% were CD3^+^ in nasal explants and 45% of the EAV-positive cells were CD172a^+^ and 15% were CD3^+^ in nasopharyngeal explants. Only a small percentage of EAV-positive cells were IgM^+^. This study validates the usefulness of a polarized mucosal explant system and shows that CD172a^+^ myeloid cells and CD3^+^ T lymphocytes represent important EAV-target cells in the respiratory mucosa.

## Introduction

The respiratory mucosa consists of the epithelium and the underlying connective tissue or *lamina propria* that are separated by a firm barrier, the basement membrane (BM). It lines the respiratory tract, including the nasal cavity, the nasopharynx, the larynx, the trachea and the bronchial tree and represents the first line of defense against respiratory infections. However, several pathogens, including equine arteritis virus (EAV) [1], use the respiratory tract as a portal of entry to invade the host. At the level of the respiratory mucosa, the mechanisms of replication and invasion of many of these pathogens are largely unknown. In vivo experiments are difficult to perform due to their costs and ethical constraints. Recently, Glorieux et al., Vandekerckhove et al., and Steukers et al. developed ex vivo explant models using gauzes to cultivate respiratory mucosae of pigs [2], horses [3] and cattle [4], respectively. These models were successfully used to get better insight into the invasion mechanisms of alphaherpesviruses [5]. However, the impossibility to inoculate the mucosa solely at the epithelial side was a limitation. In fact, inoculation of equine explants cultivated on gauzes with pseudorabies virus (PRV) resulted in infection of endothelial cells while the epithelium above them was unaffected [6]. Therefore, the author concluded that infection occurred via the side regions of the explants [6]. In line with this, when equine explants cultivated on gauzes are experimentally infected with a European EAV strain (*08P178*)*,* the majority of infected cells are localized in the connective tissue mainly at the bottom and lateral sides of the explants. In an in vivo situation, viruses have to overcome the epithelial layer before invading the host. In the explant system using only gauzes, all sides of the mucosal explants become exposed to the virus and this barrier phenomenon is, therefore, lost. As a consequence, early pathogenic events of certain respiratory agents, such as EAV, may be quite different in these ex vivo models compared to those in the in vivo situation. Therefore, it was necessary to create a model where the virus could only be delivered at the apical side. The present study describes a polarized agarose embedded explant model which allows cultivation of equine nasal and nasopharyngeal mucosae in a semi-closed system where only the epithelium side of the explant is exposed to virus upon inoculation. Agarose is a linear galactose polymer obtained by purification of agar [7] and it is frequently used as an alternative to agar in viral plaque assays [8] and microbiological investigations [9]. In the present study, agarose embedded respiratory mucosal explants were tested for morphometric integrity and cell viability. Additionally, this polarized ex vivo model was used to investigate EAV replication sites and to identify the mucosal target cells.

## Material and methods

### Collection of nasal and nasopharyngeal mucosae and preparation of agarose embedded explant cultures

Nasal septum, nasopharynx and blood from seven horses of both genders and different ages (3-6 years old) were collected at the slaughterhouse as previously described [3]. The collected tissues were immediately transported to the laboratory in transport medium: phosphate buffered saline (PBS) supplemented with 10 μg/mL gentamicin (Invitrogen, Ghent, Belgium), 1 mg/mL streptomycin (Certa, Braine l’Alleud, Belgium), 1 mg/mL kanamycin (Sigma, Bornem, Belgium), 1000U/mL penicillin (Continental Pharma, Puurs, Belgium) and 5 μg/mL fungizone (Bristol-Myers Squibb, New York, USA). The mucosae were stripped from the underlying layers and divided into equal fractions of 2 cm^2^. The nasal explants were cultured on gauzes for 24 h in 50% D-MEM (Invitrogen)/50% F12 (Invitrogen) serum-free medium supplemented with 1 μg/mL gentamicin, 0.1 mg/mL streptomycin and 100U/mL penicillin. The nasopharyngeal explants were cultured on gauzes for 24 h in 50% D-MEM/50% RPMI (Invitrogen) serum-free medium supplemented with 1nM Ca^++^, 1 μg/mL gentamicin, 0.1 mg/mL streptomycin and 100U/mL penicillin.

Six-well culture dishes were pre-seeded with rabbit kidney cells (RK13) and incubated at 37°C in the presence of 5% CO_2_ until confluency was reached. Afterwards, RK13 medium was replaced with 3 mL of a solution containing 50% of sterile 3% agarose (low temperature gelling; Sigma) and 50% of 2X medium (50% 2X D-MEM/50% 2X F12 supplemented with 2 μg/mL gentamicin, 0.2 mg/mL streptomycin and 200U/mL penicillin for nasal explants; 50% 2X D-MEM/50% 2X RPMI supplemented with 2nM Ca^++^, 2 μg/mL gentamicin, 0.2 mg/mL streptomycin and 200U/mL penicillin for nasopharyngeal explants). Once the agarose layer solidified, 1.5 cm^2^ of stripped mucosa was placed on top of it with the epithelium upwards. Additional agarose was added until the lateral surfaces of the mucosa were fully occluded. Finally, to avoid drying out during incubation (37°C and 5% CO_2_), the nasal and nasopharyngeal mucosae were covered with a tiny film of nasal or nasopharyngeal serum-free medium, respectively. Nasal and nasopharyngeal mucosae collected from three horses were used to examine the structural integrity and viability of the agarose embedded explants. Upper respiratory mucosae of the remaining four animals were included to investigate the replication and to identify the target cells of EAV *08P178* in the agarose embedded explants. To exclude previous EAV infection of the donor animals, blood samples collected at the slaughterhouse were tested for EAV specific antibodies by means of a complement-dependent virus neutralization (VN) test and an immunoperoxidase monolayer assay (IPMA) as previously described [1]. Further, to exclude recent EAV infection, 20% suspensions of nasal and nasopharyngeal mucosae were processed for virus titration on RK13 cells, immediately after collection at the slaughterhouse.

### Morphometric analysis

At 0, 24, 48 and 72 h of cultivation, nasal and nasopharyngeal explants were fixed in phosphate buffered 3.5% formaldehyde solution for 24 h. After fixation, the tissues were further processed with an automated system (Shandon Citadel Tissue Processor, 420 D) and embedded in paraffin. Afterwards, haematoxylin-eosin (HE), reticulin and Van Gieson stainings were performed. At each time point, the effect of ex vivo cultivation on the mucosa architecture was evaluated in five randomly selected fields. The structure and thickness of the epithelium were analyzed in the HE stained sections. The thickness and the continuity of the basement membrane were evaluated in the reticulin stained sections. The relative amounts of collagen and nuclei were calculated in a defined region of interest from five randomly chosen fields in the Van Gieson stained sections. All measurements and calculations were performed using the Cell F Software linked to a BX61 light microscope (Olympus Optical Co., Hamburg, Germany).

### Evaluation of cell viability

The effect of ex vivo cultivation on cell viability was evaluated using an in-situ cell death detection kit (Roche Diagnostics Corporation, Basel, Switzerland), based on terminal deoxynucleotidyl transferase mediated dUTP nick end labeling (TUNEL). The TUNEL test was performed according to the manufacturer’s guidelines on cryosections preserved in methocel^®^ (Sigma). In both epithelium and connective tissues, the percentage of TUNEL-positive cells was assessed in five randomly chosen fields of 100 cells each.

### Validation of the impermeability of agarose to virus particles

To confirm the agarose impermeability to virus particles, a preliminary experiment with fluorescent microspheres was performed. Briefly, a dual-chamber system was created by placing a transwell insert (pore size 0.4 μm, 12 mm of diameter; Millipore corporation, Bedford, MA, USA) filled with 3 mL of 50% agarose (3%) and 50% 2X medium into a culture dish of a six-well plate. Three mL of PBS were added to the bottom compartment. One mL of PBS containing 10^7.6^ streptavidin-labeled microspheres (40nm) (Invitrogen) with slightly smaller dimensions compared to EAV particles (50-100nm) [10] was brought to the upper compartment, on top of the agarose layer. Zero, 1, 12, 48, 72 and 96 h later, 50 μL of fluid was collected from both the upper and the bottom compartments and analyzed with a fluoroskan ascent FL (Thermo lab system, Altrincham, GB, UK).

To verify that the virus did not cross the agarose layer during the experimental inoculation, wells used to culture agarose embedded EAV-inoculated explants were pre-seeded with RK13 cells. After collection of the explants, the agarose layer was gently removed from the wells and the underlying RK13 monolayer was washed, air dried and submitted for indirect immunohistochemistry (IHC). Briefly, the monolayers were fixed with 4% paraformaldehyde for 10 min at room temperature, washed twice with PBS and further incubated with methanol supplemented with 0.1% H_2_O_2_. The monolayers were subsequently incubated for 1 h with mouse monoclonal antibody (mAb) 17D3 (1:100), specific for the nucleocapsid (N) protein of EAV (VMRD, Pullman, USA) and with a peroxidase-labeled goat anti-mouse IgG immunoglobulin (Molecular Probes, Oregon, USA) (1:500). Viral antigen-positive RK13 cells were visualized with 0.05M of 3-amino-9-ethylcarbazole in acetate buffer (pH5) supplemented with 0.05% H_2_O_2_ and detected by light microscopy (Olympus Optical Co.).

### Virus inoculation

After validation of the agarose embedded explant system, the model was used to investigate EAV replication and to identify the target cells. Nasal and nasopharyngeal agarose embedded mucosal explants of three horses were inoculated with 1 mL of medium containing 10^7.6^ 50% endpoint tissue culture infectious dose (TCID_50_) of a European respiratory strain of EAV (isolated in Belgium, designated *08P178*, 4^th^ passage on RK13 cells) [GenBank: JN25761], [1]. The agarose embedded explants of a fourth animal were mock-inoculated with 1 mL of medium. After 1 h of incubation at 37°C and 5% CO_2_, the inoculum was removed, the explants were gently washed twice and further incubated with fresh medium. At 0, 24, 48 and 72 hpi, specimens were collected for quantification and identification of EAV infected cells by double immunofluorescence stainings (IF).

### Identification of EAV infected cells

To quantify and identify individual EAV-positive cells, a double immunofluorescence staining was performed. For each donor animal and at each collection time, sixty 8 μm-thick cryosections of nasal mucosa and sixty 8 μm-thick cryosections of nasopharyngeal mucosa were cut and fixed in methanol at -20°C for 20 min. For each tissue, twenty cryosections were stained for each cell surface marker separately. In the first step, optimal dilutions of mAb DH59B (VMRD), UC F6G-3 (California University, Davis, USA) or 1.9/3.2 (VMRD) were used as markers for CD172a^+^ cells (monocyte lineage), CD3^+^ cells (pan T lymphocytes) and IgM^+^ cells (B lymphocytes), respectively. Afterwards, slides were incubated with Texas Red^®^-labeled goat anti-mouse IgG antibodies (Molecular Probes). In the second step, EAV nucleocapsid (N) protein was visualized with mAb 17D3 (VMRD), directly labeled with Zenon^®^ Alexa Fluor^®^ 488 Mouse IgG1 Labeling Kit, according to the manufacturer’s instructions. As a negative control, sections of the mock-inoculated nasal mucosa were stained following the aforementioned protocol. In addition, ten extra sections of inoculated nasal mucosa were incubated with CD172a^+^ as primary antibody and Texas Red^®^-labeled goat anti-mouse IgG antibodies as secondary antibody. In a second step, the glass was further incubated with an irrelevant IgG1 directly labeled with Zenon^®^ Alexa Fluor^®^ 488 Mouse IgG1 Labeling Kit. All antibodies were diluted in PBS and incubated for 1 h at 37°C. The nuclei were counterstained with Hoechst 33342 (Molecular Probes) for 10 min followed by a further fixation step of 5 min with 4% paraformaldehyde. The number of viral-antigen positive cells was determined in an area of 8 mm^2^ for each nasal and nasopharyngeal mucosae. Further, at each time point, the percentage of double positive cells (EAV-surface marker^+^) was calculated on the total amount of EAV-positive cells counted in the 20 sections stained for that specific cell marker. The samples were analyzed with a confocal microscope (Leica TCS SP2 Laser scanning spectral confocal system, Leica microsystems GmbH, Wetzlar, Germany).

### Statistical analysis

Morphometric parameters and viability were analyzed using Prism software to evaluate the variance (ANOVA). The data are presented as means ± standard deviation of triple independent experiments. The results were considered to be significantly different when *p* < 0.05.

## Results

### Morphometry

**Figure 1:**
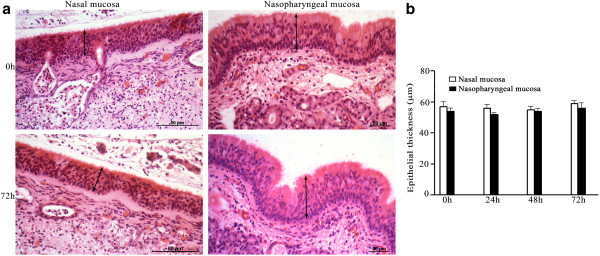
**Evaluation of the continuity (a) and the thickness (b) of nasal and nasopharyngeal epithelium (HE-staining). **The epithelial layer remained intact during the entire experiment (72 h). The thickness (indicated by arrow) of the epithelial layer did not show significant differences over time. Data are represented as means ± S.D. of triplicate independent experiments.

**Figure 2:**
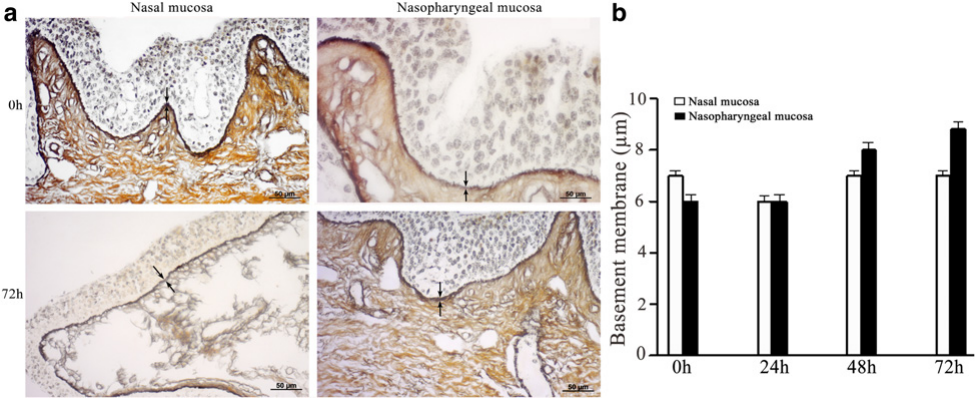
**Evaluation of the continuity (a) and the thickness (b) of nasal and nasopharyngeal basement membrane (reticulin-staining). **The collagen type III reticular fiber layer of the basement membrane (lamina reticularis) (indicated by arrows) remained continuous during the entire experiment (72 h). No significant differences in the thickness of the basement membrane were found at any time point. Data represent means ± S.D. of triplicate independent experiments.

In both nasal and nasopharyngeal mucosal explants, the architecture of the epithelium (Figure 1a), and the continuity of the basement membrane (Figure 2a) were preserved throughout the entire experiment. The thickness of the epithelium and the basement membrane did not significantly change over time (Figures 1b and 2b, respectively).

**Figure 3:**
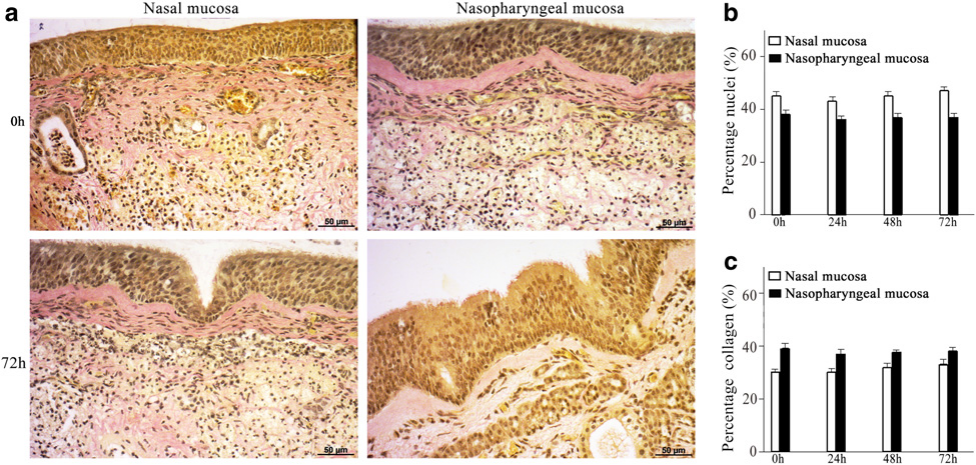
**Percentage of cells (a-b) and collagen (a-c) in nasal and nasopharyngeal mucosae (Van Gieson-staining). **By giving different colors to collagen and nuclei (setting a threshold), relative amounts of collagen and nuclei were measured during cultivation (72 h) by means of light microscopy. No significant differences in the percentage of cells and collagen were found at any time point. Data represent means ± S.D. of triplicate independent experiments.

In both nasal and nasopharyngeal mucosae, the relative amounts of nuclei (Figures 3a, 3b) and collagen (Figures 3a and 3c) were conserved over time.

### Viability

The effect of ex vivo culture on explant viability is given in Table 1. The nasal and nasopharyngeal mucosal explants show a slight, not statistically significant, increase in the number of apoptotic epithelial and stromal cells at 72 h of cultivation.

**Table 1:** Percentage of TUNEL-positive cells in the epithelium and underlying connective tissue.

	**Percentage of TUNEL-positive cells at…h of cultivation**				
		**0**	**24**	**48**	**72**
Nasal mucosa	epithelium	0.2 ± 0.4	0.6 ± 0.6	0.4 ± 0.2	0.4 ± 0.8
	lamina *propria*	0.8 ± 0.7	1.3 ± 1.0	3.0 ± 0.7	4.9 ± 2.7
Nasopharyngeal mucosa	epithelium	0.4 ± 0.3	0.8 ± 1.0	0.7 ± 1.3	0.7 ± 0.5
	lamina *propria*	0.5 ± 0.8	1.7 ± 1.8	3.0 ± 0.4	5.5 ± 1.9

Percentage of TUNEL-positive cells was used as a parameter to determine the effect of in vitro culture on the viability of equine nasal and nasopharyngeal agarose embedded mucosal explants. No significant differences in the occurrence of apoptosis were found at any time point. Data are represented as means ± S.D. of triplicate independent experiments.

### Ability of agarose to restrain virus particles

The bead assay, as specified in the material and methods, indicates the ability of agarose to restrain virus particles. None of the samples collected from the bottom compartment of the dual-chamber system contained streptavidin-labeled microspheres at any time point. Microspheres remained present in the fluid collected from the upper compartment up to 96 hpi. In the absence of agarose, microspheres were readily observed in the lower compartment.

In addition, IHC staining performed on the RK13 monolayer seeded underneath the EAV-inoculated agarose embedded explants did not reveal EAV-positive cells at any time point.

### Quantification and identification of EAV-antigen positive cells in agarose embedded explants

**Figure 4:**
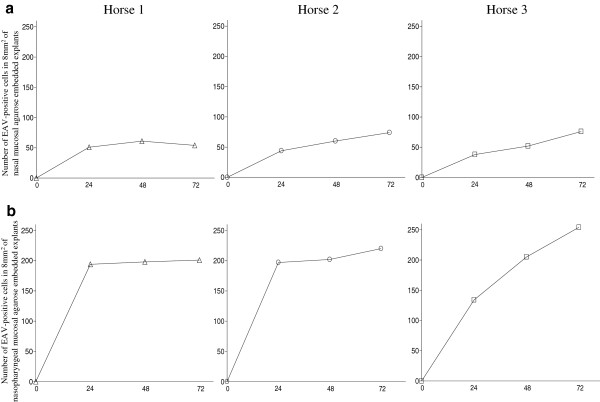
**Number of antigen-positive cells/8 mm**^**2 **^**in agarose embedded nasal (a) and nasopharyngeal (b) explants. **Of each donor animal and at each collection time, sixty 8 μm-thick cryosections of nasal mucosa and sixty 8 μm-thick cryosections of nasopharyngeal mucosa were cut and fixed in methanol. EAV nucleocapsid (N) protein was visualized with mAb 17D3, directly labeled with Zenon^® ^Alexa Fluor^® ^488 Mouse IgG1 Labeling Kit, according to the manufacturer’s instructions. The nuclei were counterstained with Hoechst 33342. The number of viral-antigen positive cells was determined in an area of 8 mm^2 ^for each nasal and nasopharyngeal mucosae. The number of EAV-positive cells increased in time with a mean of 68 in 8 mm^2 ^of nasal mucosa and 225 in 8 mm^2 ^of nasopharyngeal mucosa at 72 hpi. In particular, 54, 74 and 76 antigen-positive cells/8 mm^2 ^were counted in nasal explants and 201, 220 and 254 EAV-positive cells/8 mm^2 ^were quantified in nasopharyngeal explants of horse 1, 2 and 3, respectively.

**Figure 5:**
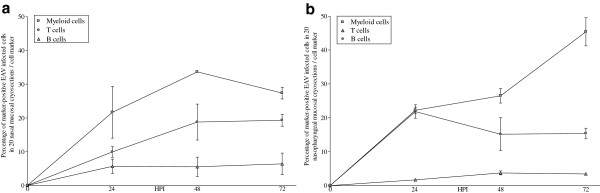
**Identification of EAV-positive leukocytes in agarose embedded nasal (a) and nasopharyngeal (b) explants. **Lines show the means ± S.D. of triplicate independent experiments.

**Figure 6:**
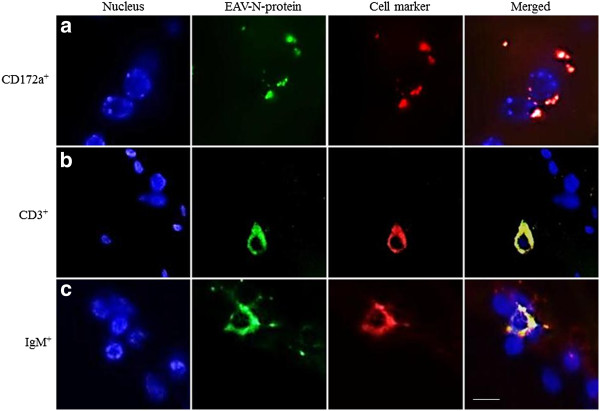
**Representative confocal photomicrographs illustrating viral tropism for leukocyte subpopulations. **Two different types of EAV-positive cells can be distinguished: big cells with a big round or oval nucleus surrounded by large cytoplasmic extensions mostly CD172a^+ ^(**a**) and rather small cells with a big round or oval nucleus surrounded by a small rim of cytoplasm mostly CD3^+ ^or IgM^+ ^(**b**-**c**). The viral antigens could be found in the form of small dots (**a**) or in the form of large masses occupying most of the cytoplasm (**b**-**c**). Scale bar represents 10 μm.

Samples included as negative control did not show EAV-positive cells at any time point. The number of EAV-positive cells increased in time with a mean of 68 in 8 mm^2^ of nasal mucosa and 225 in 8 mm^2^ of nasopharyngeal mucosa at 72 hpi. In particular, 54, 74 and 76 antigen-positive cells/8 mm^2^ were counted in nasal explants (Figure 4a) and 201, 220 and 254 EAV-positive cells/8 mm^2^ were quantified in nasopharyngeal explants (Figure 4b) of horse 1, 2 and 3, respectively. Specifically, in nasal mucosa, 22, 34 and 27% of EAV-positive cells were identified as CD172a^+^ at 24, 48 and 72 hpi, respectively. The percentage of EAV infected cells positive for CD3 increased linearly from 10 (24 hpi) to 19% (48 and 72 hpi) during the experiment (Figure 5a). In the nasopharyngeal mucosal explants, the percentage of EAV infected cells positive for CD172a increased from 22 (24 hpi) to 26 (48 hpi) and 45% (72 hpi) over time, while the percentage of EAV infected CD3 cells reached 22% at 24hpi and decreased to 15% afterwards (48 and 72 hpi) (Figure 5b). At 24 hpi, EAV-positive cells were mainly localized in the upper layers of both nasal and nasopharyngeal mucosae and, at later time points (48 and 72 hpi), were scattered all over the connective tissue. In general, individual EAV-infected cells were mostly found under the basement membrane with a sporadic presence in between epithelial cells. In addition, in nasopharyngeal explants individual EAV-positive cells were frequently localized in the parafollicular area. Viral antigen-positive cells were not found at the cutting edges of the agarose embedded explants. Intracellularly, viral antigens were seen in the cytoplasm (Figure 6), in the form of small dots (Figure 6a) but mostly in the form of large masses occupying most of the cytoplasm (Figures 6b and c).

## Discussion

The mucosa of the upper respiratory tract (URT) represents the first line of defense against respiratory pathogens but often also serves as a portal of entry for several microorganisms, whether or not causing generalized infection. The principal viral agents responsible for respiratory disorders in horses are equine herpesvirus-1 (EHV-1) and equine herpesvirus-4 (EHV-4) [11], equine arteritis virus (EAV) [12] and equine influenza virus [13]. A previous system used by Vandekerckhove et al. allowed cultivating four representative tissues of the equine URT up to 96 h without significant changes in morphology and viability [3]. In that model, the explants were cultivated on gauzes allowing nutrients to penetrate through all sides of the mucosa. Because of the small size of the explants (0.5 cm^2^, few mm thick) and the open edges, nutrients have free access to the inner parts of the tissues. In this system, pathogens, just like nutrients, do not only enter the mucosa at the upper epithelium side, but also at the edges through the open spaces of the connective tissue. In fact, inoculation of such explant systems with PRV [6] or EAV *08P178* (our own studies, not published) resulted in an extensive spread of the virus, mainly at the bottom and lateral sides of the mucosal explants. Therefore, in order to better mimic the in vivo situation, we established a new model in which, through agarose embedded explants, a polarized system was obtained so that pathogens could only enter through the upper epithelium. The results of morphometric analysis of the epithelium, basement membrane and connective tissue and of TUNEL assays demonstrate that the agarose system allows cultivating explants up to 72 h without substantial alterations in the morphology and the viability of the tissues. Further, an assay based on streptavidin-labeled microspheres together with the absence of EAV-antigens in RK13 cells seeded underneath the agarose layer in which inoculated explants were embedded and the lack of viral antigen-positive cells at the cutting edges of the agarose embedded explants, demonstrate that the access of the pathogens in the system was restricted to the apical side of the epithelium.

We recently showed that, upon in vivo oro-nasal inoculation, EAV *08P178* starts its replication in nasal and nasopharyngeal regions and lungs [1]. Therefore, to gain more insight into the early phase of EAV respiratory infection, kinetic studies were performed by inoculating the agarose embedded nasal and nasopharyngeal mucosal explants with EAV *08P178*. Currently, little is known about the early phases of EAV pathogenesis such as (i) which strategy EAV uses to invade the host, (ii) what are the first cells to be infected at the entry site or (iii) how blood vessels are reached to disseminate the virus throughout the body. The current study showed that in the explant model, the majority of EAV-positive cells were CD172a^+^ myeloid cells followed by CD3^+^ T-lymphocytes, whereas only a small percentage were IgM^+^ B-lymphocytes. Previously, we speculated that, at the level of the URT, EAV hijacks mononuclear leukocytes to penetrate the BM and evade the immune response [1]. In possible support of this, the present study shows a high percentage of EAV-infected CD172a^+^ cells. CD172a was expressed on equine monocytes, macrophages, dendritic cells (DC) and granulocytes. DC have specific characteristics: (i) they have a major role in activating naïve and resting antigen-specific T cells [14], (ii) they are migratory cells, unlike differentiated macrophages [15] and (iii) they are abundantly present within the airway epithelium [16] forming a network of antigen presenting cells in the respiratory mucosa [17,18]. DC play a central role in initiating an immune response against infecting agents [19] but may also contribute to the pathogenesis and the spreading of several respiratory pathogens as shown for human [20] and simian [21] immunodeficiency viruses, cytomegalovirus [22] and *Mycobacterium tuberculosis*[23]. Hence, it is tempting to speculate that DC could play an important role in the early phases of EAV pathogenesis. This theory could corroborate the findings of Del Piero that described EAV-antigens within stromal dendrite-like cells of lymph node sinuses and spleen [12]. On the contrary, since the main function of myeloid cells is to capture antigens, CD172a^+^ cells may be positive for the EAV nucleocapsid protein due to phagocytosis rather than infection.

It is generally believed that EAV has a tropism mainly for the monocyte/macrophage population. However, Castillo-Olivares et al. showed that EAV-infected PBMC are negative for the human monocyte/granulocyte marker L1 (calprotectin) [24]. In the present study, both T-lymphocytes and cells of the monocyte/macrophage lineage were found to be infected with EAV. Other studies have also demonstrated that EAV can infect CD3^+^ T lymphocytes in PBMC in vitro [25,26].

In the present study, 37 to 64% of EAV-positive cells were identified. Since EAV has a wide tropism [27], the percentage of non-identified EAV-positive cells may be epithelial cells, endothelial cells, mesenchymal stromal cells, and natural killer cells. In fact, in vivo, EAV-antigens were demonstrated within the cytoplasm of endothelial cells, myometrial and cardiac myocytes, chorionic mesenchymal stromal cells and epithelial cells such as alveolar pneumocytes, enterocytes, adrenal cortical cells, trophoblast, thymus stroma, and renal tubular cells [27]. Therefore, it will be interesting to further identify the remaining EAV-positive cells in future experiments using the agarose explant system.

Although polarized mucosal explants are much more physiological than non-polarized ones and in vitro cell cultures, they are only suitable to study the early stages of the pathogenesis of viruses that use the respiratory tract as a portal of entry. It must be kept in mind that the mucosal explants not fully represent the whole scope of virus-animal interactions since the inflammatory processes including invasion of blood elements are absent. However, preliminary results obtained with nasal mucosa from experimentally EAV-inoculated ponies were very similar to those of the present in vitro study, confirming the relevance of the polarized mucosal explant system. In conclusion, the present study demonstrates that the agarose embedded explant model represents a valid tool to study some aspects of the early steps in the pathogenesis of equine respiratory viruses such as EAV and that mucosal CD172a^+^ myeloid cells and CD3^+^ T lymphocytes represent important target cells for EAV.

## Competing interests

The authors declare that they have no competing interests.

## Authors’ contributions

SV set up the study design, carried out the optimization of the organ culture as well as the infection experiments and the processing of all samples, performed the statistical analysis and drafted the manuscript. WVdB participated in the analysis and the interpretation of all the morphological results. HF took part in the analysis and the interpretation of all the immunofluorescence results. AS assisted in the set up of both experiments and sampling. HN coordinated the study and participated in its design. All authors read and approved the final manuscript.

## References

[B1] Vairo S Vandekerckhove A Steukers L Glorieux S Van den Broeck W Nauwynck H Clinical and virological outcome of an infection with the Belgian equine arteritis virus strain *08P178* Vet Microbiol 2012 157 333 344 10.1016/j.vetmic.2012.01.014 22306037

[B2] Glorieux S Van den Broeck W van der Meulen K Van Reeth K Favoreel H Nauwynck H In vitro culture of porcine respiratory nasal mucosa explants for studying the interaction of porcine viruses with the respiratory tract J Virol Methods 2007 142 105 112 10.1016/j.jviromet.2007.01.018 17324473

[B3] Vandekerckhove A Glorieux S Van den Broeck W Gryspeerdt A Van der Meulen K Nauwynck H In vitro culture of equine respiratory mucosa explants Vet J 2009 181 280 287 10.1016/j.tvjl.2008.03.027 18539059

[B4] Steukers L Vandekerckhove A Van den Broeck W Glorieux S Nauwynck H Comparative analysis of replication characteristics of BoHV-1 subtypes in bovine respiratory and genital mucosa explants: a phylogenetic enlightenment Vet Res 2011 42 33 10.1186/1297-9716-42-33 21324115 PMC3050707

[B5] Steukers L Glorieux S Vandekerckhove A Favoreel H Nauwynck H Diverse microbial interactions with the basement membrane barrier Trends Microbiol 2012 20 147 155 10.1016/j.tim.2012.01.001 22300759 PMC7127156

[B6] Vandekerckhove A Equine nasal mucosal explants, a valuable tool to study early events of the pathogenesis of equine herpesvirus infections PhD thesis 2011 Merelbeke, Belgium: Gent University, Virology Immunology and Parassitology Department

[B7] Araki C Wolfrom ML Carbohydrate chemistry of substances of biological interest Fourth International Congress of Biochemistry, symposium 1. 1–6 September 1995 1958 Vienna: Pergamon Press 1 15

[B8] De Maeyer E Schonne E Starch gel as an overlay for the plaque assay of animal viruses Virology 1964 24 13 18 10.1016/0042-6822(64)90142-4 14208896

[B9] Hamilton A Robinson C Sutcliffe C Slater J Maskell J Davis-Poynter N Smith K Waller A Harrington1 J: Mutation of the maturase lipoprotein attenuates the virulence of *streptococcus equi* to a greater extent than does loss of general lipoprotein lipidation Infect Immun 2006 74 6907 6919 10.1128/IAI.01116-06 17015455 PMC1698103

[B10] Bürki F Hofer A Nowotny N Objective data plead to suspend import-bans for seroreactors against equine arteritis virus except for breeder stallions J Appl Anim Res 1992 1 31 42 10.1080/09712119.1992.9705906

[B11] Van Maanen C Equine herpesvirus 1 and 4 infections: an update Vet Q 2002 24 58 78 12095082

[B12] Del Piero F Equine viral arteritis Vet Pathol 2000 37 287 296 10.1354/vp.37-4-287 10896389

[B13] Mumford E Traub-Dargatz J Salman M Collins J Getzy D Carman J Monitoring and detection of acute viral respiratory tract disease in horses J Am Vet Med Assoc 1998 213 385 390 9702229

[B14] Ingulli E Mondino A Khotuts A Jenkins MK In vivo detection of dendritic cell antigen presentation to CD4­T cells J Exp Med 1997 185 2133 2141 10.1084/jem.185.12.2133 9182685 PMC2196354

[B15] Lipscomb MF Masten BJ Dendritic cells: immune regulators in health and disease Physiol Rev 2002 82 97 130 11773610 10.1152/physrev.00023.2001

[B16] Holt PG Stumbles PA Characterization of dendritic cell populations in the respiratory tract J Aerosol Med 2000 13 361 367 10.1089/jam.2000.13.361 11262442

[B17] Holt PG Schon-Hegard MA Phillips MJ McMenamin PG Ia-positive dendritic cells form a tightly meshed network within the human airway epithelium Clin Exp Allergy 1989 19 597 601 10.1111/j.1365-2222.1989.tb02752.x 2688847

[B18] Schon-Hegrad MA McMenamin OJ Holt PG Studies on the density distribution and surface phenotype of intraepithelial class II major histocompatibility complex antigen (la)-bearing dendritic cells (DC) in the conducting airways J Exp Med 1991 173 1345 1356 10.1084/jem.173.6.1345 2033368 PMC2190835

[B19] Banchereau J Steinman RM Dendritic cells and the control of immunity Nature 1998 392 245 252 10.1038/32588 9521319

[B20] Ayehunie S Garcia-Zepeda EA Hoxie JA Horuk R Kupper TS Luster AD Ruprecht RM Human immunodeficiency virus-1 entry into purified dendritic cells through CC and CXC chemokine coreceptors Blood 1997 90 1379 1386 9269754

[B21] Hu J Pope M Brown C O’Doherty U Miller CJ Immunophenotypic characterization of SIV-infected dendritic cells in cervix vagina and draining lymph nodes of rhesus monkeys Lab Invest 1998 78 435 451 9564888

[B22] Hahn G Jores R Mocarski ES Cytomegalovirus remains latent in a common precursor of dendritic and myeloid cells Proc Natl Acad Sci U S A 1998 95 3937 3942 10.1073/pnas.95.7.3937 9520471 PMC19941

[B23] Smith J *Mycobacterium tuberculosis* pathogenesis and molecular determinants of virulence Clin Microbiol Rev 2003 16 463 496 10.1128/CMR.16.3.463-496.2003 12857778 PMC164219

[B24] Castillo-Olivares J Tearle J Montesso F Westcott D Kydd J Davis-Poynter N Hannant D Detection of equine arteritis virus (EAV)-specific cytotoxic CD8^+^ T lymphocyte precursors from EAV-infected ponies J Gen Virol 2003 84 2745 2753 10.1099/vir.0.19144-0 13679609

[B25] Go Y Bailey E Cook D Coleman S Macleod J Chen K Timoney P Balasuriya U Genome-wide association study among four horse breeds identifies a common haplotype associated with in vitro CD3^+^ T cell susceptibility/resistance to equine arteritis virus infection J Virol 2011 85 13174 13184 10.1128/JVI.06068-11 21994447 PMC3233183

[B26] Go Y Zhang J Timoney P Cook R Horohov D Balasuriya U Complex interactions between the major and minor envelope proteins of equine arteritis virus determine its tropism for equine CD3^+^ T lymphocytes and CD14^+^ monocytes J Virol 2010 84 4898 4911 10.1128/JVI.02743-09 20219931 PMC2863813

[B27] Del Piero F IVIS.org Equine viral arteritis: signs lesions pathogenesis and diagnoses Proceedings of the Annual Meeting of the American College of Veterinary Pathologists and American Society for Veterinary Clinical Pathology: 2-6 December 2006 2006 Tucson, Arizona:

